# Development and Validation of Ambulosono: A Wearable Sensor for Bio-Feedback Rehabilitation Training

**DOI:** 10.3390/s19030686

**Published:** 2019-02-08

**Authors:** Taylor Chomiak, Abhijot Singh Sidhu, Alexander Watts, Luke Su, Brian Graham, Joshua Wu, Suzanne Classen, Brian Falter, Bin Hu

**Affiliations:** 1 Department of Clinical Neurosciences, Hotchkiss Brain Institute, University of Calgary, Calgary, AB T2N 4N1, Canada; sidhuas@ucalgary.ca (A.S.S.); awatts@ualberta.ca (A.W.); 2 Alberta Children’s Hospital Research Institute, Calgary, AB T2N 4N1, Canada; 3 Cumming School of Medicine Bachelor of Health Sciences, University of Calgary, Calgary, AB T2N 4N1, Canada; 4 Faculty of Medicine and Dentistry, University of Alberta, T6G 2R7, Edmonton, AB, Canada; 5 Softwisys Professionals Inc., Calgary, AB T2L 2K7, Canada; luke.su@softwisys.com (L.S.); brian.graham@softwisys.com (B.G.); joshua.wu@softwisys.com (J.W.); suzanne.classen@softwisys.com (S.C.); 6 Electronic Innovation Inc., Calgary, AB T2W 1Z7, Canada; bfalter@telus.net

**Keywords:** wearable, sensors, Ambulosono, rehabilitation, gait

## Abstract

Wearable technology-based measurement systems hold potential for the therapeutic and rehabilitation management of patients with various chronic diseases. The purpose of this study was to assess the accuracy and test–retest reliability of a new-generation wearable sensor-based system, dubbed Ambulosono, for bio-feedback training. The Ambulosono sensor system was cross-validated by comparing its functionality with the iPod touch (4th generation) sensor system. Fifteen participants underwent a gait test to measure various gait parameters while wearing both the iPod-based and Ambulosono sensors simultaneously. The physically measured values (i.e., the true values) of step length, distance traveled, velocity, and cadence were then compared to those obtained via the two-sensor systems using the same calculation algorithms. While the mean percentage error was <10% for all measured parameters, and the intra-class correlation coefficient revealed a high level of agreement between trials for both sensor systems, it was found that the Ambulosono sensor system outperformed the iPod-based system in some respects. The Ambulosono sensor system possessed both reliability and accuracy in obtaining gait parameter measurements, which suggests it can serve as an economical alternative to the iPod-based system that is currently used in various clinical rehabilitation programs.

## 1. Introduction

Advancements in technology and manufacturing have expanded the application of new wearable technology-based measurement systems for the detection and monitoring of a range of functional aspects of disease under a variety of conditions [1,2,3,4,5,6]. The comprehensive characterization and long-term monitoring of patients by wearable sensor systems offers a practical tool to reduce the standard deviation of clinical endpoints and minimize inter-rater variability of clinical assessments [2]. These wearable technologies have the potential to improve diagnostic precision and therapeutic management of patients, as well as the quality of life for patients and their families [1,2,3,4,5,6]. However, in order for wearable sensor systems to be of any practical use, objective measurements of function need to represent variables that are relevant to the condition, be amenable to interventions by clinicians and researchers, be accurately measured, and should exhibit consistency among repeated measurements [2,5].

We recently developed the Ambulosono sensor system. This system utilizes high-precision movement sensors to digitally link self-generated movements with physiologically defined sound and music cues to enable contingency-based perceptual-motor learning and anticipatory gait control [4,7]. The Ambulosono system was initially developed using the iPod touch (4th generation), which has built-in motion sensors consisting of a 3-axis Micro-Electro-Mechanical Systems (MEMS)-based gyroscope and a 3-axis accelerometer (six dimensions). MEMS technologies, in which miniaturized mechanical and electro-mechanical elements of the device are made using the techniques of microfabrication, can often far exceed their macroscale counterparts with respect to device performance. The iPod touch firmware uses proprietary fusion codes for automatic gravity and sensitivity calibration and real-time attitude angles output (pitch, roll and yaw), while our GaitReminder™ App utilizes algorithms to sample sensor output for real-time gait calculations after corrections for limb length, angular excursion, signal filtering and drift, which has shown promise for use in clinical assessments and therapeutic applications [4,7]. 

The Ambulosono system uses a multi-functional, high-precision wearable sensor to record a user’s movement responses, which are interactively cued and controlled by a rich acoustic library of verbal instructions, sound, and music files stored on a user’s mobile device [4,7]. A coordinator or therapist can then design and personalize training or assessment programs based on an individual’s history, dominant symptoms, and/or rehabilitation needs. Importantly, training modality and sensor parameters can be reprogrammed remotely based on training dosage, progress, or outcome, without the need for frequent and overt patient–therapist interaction. While the Ambulosono system has the potential to be scaled-up for high-volume clinical services and further developed into a new paradigm for non-pharmacological management of patients with unmet medical needs, an important limitation of this system is its current dependency on the iPod touch. This can make such a system not only economically less practical for large-scale healthcare solutions, but also impact its accessibility to individual patients. The goal of this study, therefore, was to develop and test an iPod-independent Ambulosono sensor system that can be linked via Bluetooth Low Energy (BLE) to a user’s mobile device running Android (Lollipop) 5.1 or iOS 8. Our results indicate that our newly developed Ambulosono system performed as well as, or in some instances better than, the iPod-based system.

## 2. Materials and Methods

### 2.1. Subjects and Data Collection

**Figure 1:**
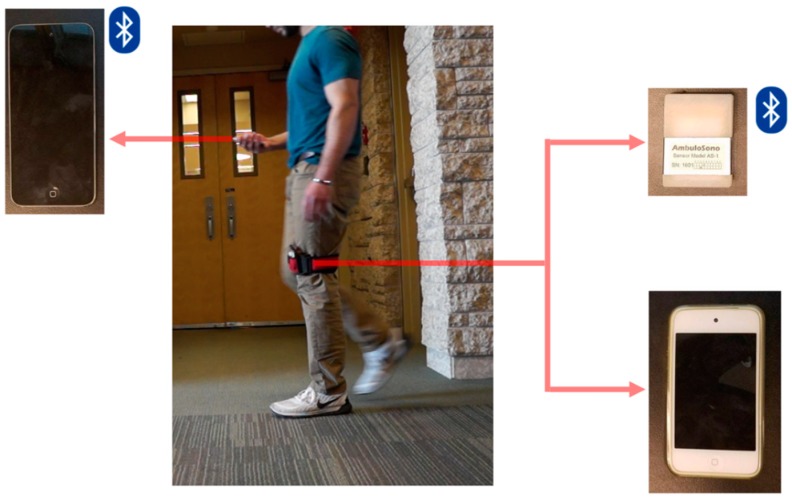
A schematic of the validation testing setup. Both sensor devices (an iPod touch 4th generation, and the Ambulosono sensor; shown on the right) were placed together into a high-performance thigh band that was positioned just above the left knee of the participant (middle panel). Both devices were used in conjunction with the GaitReminder™ App (see text for more details), and the Ambulosono sensor was connected via Bluetooth to the research investigator’s iOS device (left panel).

Ethics approval was obtained from the University of Calgary Ethics Board for Human Research, and informed written consent was obtained (REB13-0009). Fifteen healthy adult male participants (*n* = 15), with no pre-existing gait abnormalities, agreed to participate in this sensor validation study. Two mobile technologies were used, an iPod touch (4th generation) and the Ambulosono sensor. It should be noted that both devices were used in conjunction with the GaitReminder™ App, and the Ambulosono sensor was connected via Bluetooth to the research investigator’s iOS device. Prior to the validation test, the step-samples on both devices were calibrated (see Section 3.1) to correlate with each participant’s limb length (ranging from 0.86–0.94 m). Both devices were then placed together in a high-performance thigh band that was positioned just above the left knee of the participant, as shown in Figure 1. Sensor validation was conducted using the following instructions: The participants were instructed to walk, as straight as possible, down a clearly marked 30.48 m, or 100 ft, unobstructed hallway. Prior to beginning the walk, the participants were asked to stand still for several seconds until cued (verbally instructed) to begin walking at a normal self-selected pace. Once the participant reached the end of the hallway, they were again asked to stop and stand still for several seconds. Standing still before and after the walk served to delimit the data to be processed. While walking, the participants were followed and videoed using a Sony α-3000 video camera due to the limited field of view of stationary camera-based reference systems for gait assessments over longer distances [1], with which Ambulosono was designed. The video data was then used to establish the true number of steps, the time of each trial, and to verify the distance traveled via markings on the floor. In order to establish test-retest reliability, all participants took part in two trials using the exact same protocol as described above for the second trial. 

### 2.2. Data Analysis

The known distance of the hallway, the time taken to complete the trial, and the number of true steps taken were all verified by video and are referred to here as the true measurement values. These values were then used to compute the true trial velocity (meters/s), cadence (steps/min), and average step length (m). The distance was standardized for each trial/participant at 30.48 m. Velocity was calculated by dividing the known distance walked by the known time; step length was calculated by dividing the known distance traveled by the number of steps; and the cadence was calculated by dividing the known number of steps taken by the time of the walk. These values were then compared to the velocity, cadence, step length and distance measurements that were obtained by both sensor systems. After each trial, both sensors wrote a raw excel file that contained the participants’ number of steps, overall distance, and walk duration. Specifically, the step length column was averaged or summed to establish the participants’ average step length or distance traveled, respectively. Similarly, the step time column was summed to establish the participants’ walk duration. These values were then used to generate the sensor-based velocity and cadence values as described above. 

### 2.3. Statistical Analysis

All statistical analyses were computed with SPSS and GraphPad Prism. Data are expressed as mean ± SEM, unless otherwise stated. A paired *t*-test (α = 0.05) was used to compare the mean percentage error between sensors. Test-retest reliability was evaluated using a single measures intra-class correlation coefficient (ICC) with agreement:

(1) $$\frac{{MS}_{p}-{MS}_{e}}{{MS}_{p}+(k-1){MS}_{e}+\frac{k({MS}_{t}-{MS}_{e})}{n}}$$

where *MS_p_* = participants’ mean square, *MS_e_* = error mean square, *MS_t_* = trials (test sessions) mean square, *k* = number of trials, and *n* = number of participants [8]. Similarly to work published previously [9], we used the following classification to indicate the strength of the correlation coefficients: R = 0.01–0.2: negligible, R = 0.2–0.4: weak, R = 0.4–0.7: moderate, and R > 0.7: strong. The absolute mean percentage error (MPE) was calculated to establish the average percentage error by which velocity, cadence, distance, and step length values obtained from the sensors differed from the true values.

## 3. Results

### 3.1. Sensors and Gait Measurements

As we have already published [7], the iPod touch has built-in motion sensors consisting of a 3-axis MEMS-based gyroscope and a 3-axis accelerometer. The iPod touch firmware uses fusion codes for automatic gravity calibrations and real-time angle output (pitch, roll, and yaw). The Ambulosono sensor (Softwisys Professionals Inc.) was based on prototyping the Seeed Tiny BLE development board using an MPU-6050 motion processor, which provides separate Raw Accelerometer (RAW_ACCEL) data for detecting motion, and Calibrated Gyroscope (CAL_GYRO) quaternions for converting to Euler angles. For gait-cycle measurements, both the iPod touch and Ambulosono sensor were attached to the leg just above the patellofemoral joint line through the use of a high-performance thigh band [4,7], as shown in Figure 1. 

**Figure 2:**
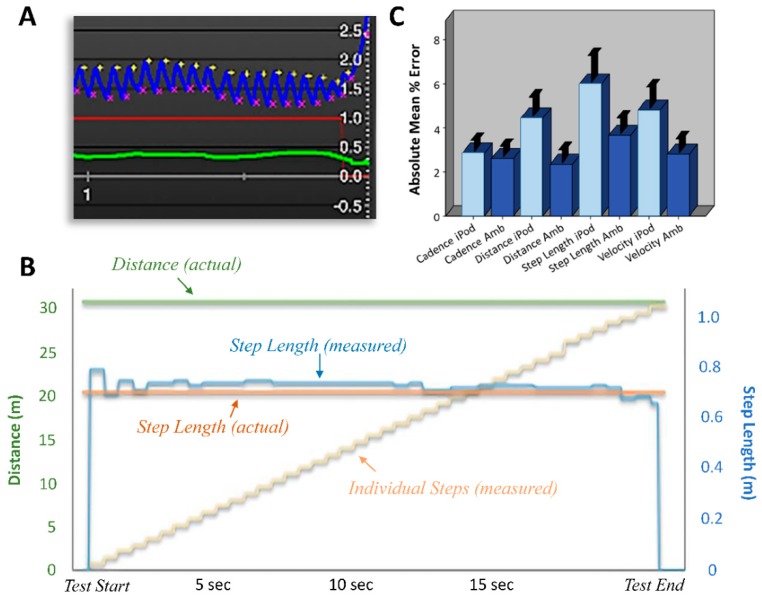
Performance of the sensor systems. (**A**) An example of a raw trace during stepping illustrating: Hip flexion (blue; with the difference between the yellow and purple cross-marks indicating the peak difference), step size (green), and whether step size is above a pre-defined threshold (red), which can be enabled and linked to music play. (**B**) An example of a comparison between average step length and the overall distance values outputted by the Ambulosono sensor system and the true values. (**C**) MPE values for all measured gait parameters for both the iPod touch sensor and the independent Ambulosono sensor system (Amb).

The software application GaitReminder™ utilizes sample sensor output for step parameter calculations based on its ability to automatically detect hip flexion in real-time [7]. The algorithm works by using circumference geometry and its concepts of radius and radians to functionally relate step amplitude with hip flexion angles by the expression:

(2) SL=Pd⋅LL

where *SL* represents step length, *Pd* represents peak hip flexion difference (see Figure 2A for an example), and *LL* represents limb length as measured from the hip-joint center to the floor. Files are then locally auto-saved, archived, encrypted, and sent to a secure server, which is accessible from anywhere in the world. 

### 3.2. Ambulosono Sensor Testing and Validation

To test the Ambulosono system, we evaluated fifteen participants (*n* = 15) who underwent a gait test where the true gait parameters were known, while wearing both the iPod-based and Ambulosono systems simultaneously (see Methods and Materials). Figure 2B illustrates the basic parameters of the sensor systems’ output, with cumulative steps and corresponding step length and total distance measures calculated in real-time, through which the average cadence and velocity are derived with elapsed time. Summary data for the average step length, distance, cadence, and velocity values obtained from the iPod and Ambulosono sensors, and for the two separate trials compared to the true values, are shown in Table 1. The mean percentage error (MPE) for all measures by either sensor were <10%, as shown in Figure 2C. However, the step length, distance, and velocity values obtained from the Ambulosono sensor tended to have a lower MPE than that of the iPod sensor (as shown in Figure 2C), with the MPE for distance measured between sensors reaching statistical significance (*t*_(14)_ = −2.33; *p* = 0.035). To quantify the test-retest reliability, the intra-class correlation coefficient (ICC) was calculated and revealed a high level of agreement between trials for all parameters with both devices, with the Ambulosono system performing slightly better (iPod ICC = 0.78–0.92 versus Ambulosono ICC = 0.82–0.92). The ICC obtained from single measures showed strong test-retest reliability for both the iPod (0.84, 95% CI 0.60–0.94; 0.78, 95% CI 0.45–0.92; 0.80, 95% CI 0.50–0.93; and 0.92, 95% CI 0.78–97 for step length; distance; cadence; and velocity, respectively, and *p* < 0.001 for each) and the Ambulosono (0.87, 95% CI 0.65–95; 0.82, 95% CI 0.54–94; 0.91, 95% CI 0.75–0.97; and 0.92, 95% CI 0.78–0.97 for step length; distance; cadence; and velocity, respectively, and *p* < 0.001 for each) sensors.

## 4. Discussion

Here, we reported that the wearable iPod-based Ambulosono device exhibited remarkable accuracy in measuring gait parameters in real-time and showed strong test-retest reliability. Furthermore, we have also shown that the wearable iPod-independent Ambulosono sensor system can perform as well as, or even outperform, the iPod-based system. 

The use of stationary systems to measure gait function, such as the GAITRite system or 3D camera-based systems, have been shown to be quite effective, and previous work using these approaches have also reported efficacy in quantifying gait in many research and rehabilitation settings [1,10,11,12,13,14,15,16]. These approaches have also been used to evaluate gait in healthy controls, with similar values in gait measurements observed to that in the present study [1,10]. While gait is often evaluated quantitatively using Vicon and GAITRite systems under specific research and rehabilitation settings, general clinical measures of gait disturbance have often relied on rating scales or semi-quantitative measures [17,18]. This may, at least in part, be due to the fact that the use of stationary gait systems may not be practical for many settings outside of the clinic. Given that common clinical motor assessment scales may be a poor indicator of ambulatory function (e.g., [18,19]), and given the relatively inexpensive cost and versatility of wearable sensors compared to stationary systems, quantifying gait with wearable sensors may indeed allow for a more objective approach to determine the functional status of patients under a variety of clinical settings [1,2,3,4,5,6,20,21,22]. However, it is important to note that patient populations can often exhibit much more variability in gait parameters than healthy controls, and as a consequence, the precision gained by using wearable sensor technology may be limited by the test-retest variability of patient performance. This is why we chose to focus this study on healthy controls, so comparisons between systems would not be confounded by patient population-specific variability. It will, however, be important for future studies to establish test-retest reliability for specific patient populations. Another limitation of this study was our relatively small sample size. While we did observe a statistically significant difference in the MPE for distance between systems, the sample size used here limited our ability to statistically discern differences in the MPE for step length and velocity between sensor systems, despite obvious differences in average values between the sensors, as shown in Figure 2C.

## 5. Conclusions

The clinical potential of body-fixed sensors has rapidly expanded due to advances in technology and data processing capabilities over the past few decades. With the ubiquitous uptake of smartphone technology, the type of gait assessment device described here may also have a real and meaningful impact on both the monitoring of gait under more naturalistic walking conditions [23], and in the therapeutic management of certain patient populations [4,24]. For example, the Ambulosono system can be considered a special form of action-sound association [4]. With the Ambulosono system, patients with Parkinson’s disease, for instance, can use step-size signals captured by the leg sensor to control the playing of highly pleasurable music during self-directed gait cycling during home-based rehabilitation training. When step amplitude becomes smaller than a pre-defined threshold, music play is automatically interrupted, thus creating a salient reminder and incentive for the user to actively engage and re-adjust their step amplitude [4]. During Ambulosono training, music delivery and gait become instrumentally conditioned and behaviorally reinforced [4]. Thus, long-term Ambulosono training may not only increase a patient’s awareness of shuffling steps at home, but it may also allow them to become capable of self-correcting step amplitude. Nevertheless, ongoing Ambulosono research from multiple testing sites indicates that the Ambulosono system has the potential to be scaled-up for high-volume clinical services and further developed into a new paradigm for non-pharmacological care management for a variety of patients with unmet medical needs. 

## Figures and Tables

**Table 1:** Summary (mean) values for Step Length, Distance, Cadence and Velocity.

Step Length (m)	Trial 1	Trial 2
Known Standard	0.71	0.71
New Sensor	0.72	0.72
iPod	0.67	0.67
**Distance (m)**		
Known Standard	30.48	30.48
New Sensor	30.13	30.06
iPod	29.28	29.18
**Cadence (steps/min)**		
Known Standard	106.76	106.09
New Sensor	107.16	108.26
iPod	106.05	108.33
**Velocity (m/s)**		
Known Standard	1.24	1.26
New Sensor	1.27	1.28
iPod	1.2	1.21
